# Reliability of a Home-Based Smartphone Balance Assessment in Healthy Middle-Aged and Older Adults

**DOI:** 10.3390/s26165219

**Published:** 2026-08-18

**Authors:** Elizabeth Coker, Anat V. Lubetzky

**Affiliations:** 1 Department of Dance, Tisch School of the Arts, New York University, New York, NY 10003, USA; 2 Department of Physical Therapy, Steinhardt School of Culture, Education, and Human Development, New York University, New York, NY 10010, USA; anat@nyu.edu

**Keywords:** standing balance, smartphone accelerometry, reliability, middle-aged, aging, home assessment

## Abstract

**Highlights:**

**What are the main findings?**

- A custom smartphone application produced good-to-excellent reliability for remote balance assessment when sway measures were averaged across three weekly home-based tests.
- Time-domain metrics, particularly mean acceleration, demonstrated the highest reliability and measurement precision, while reliability decreased as balance tasks became more challenging.

**What are the implications of the main findings?**

- Smartphone-based balance assessment can provide reliable and precise measurements without in-person supervision, supporting scalable remote monitoring.
- This approach could facilitate large-scale balance assessment, improving accessibility for middle-aged and older adults.

**Abstract:**

Smartphone accelerometry could enable longitudinal home-based balance testing, yet its reliability across tasks and outcome measures must be established. We assessed the test–retest reliability and measurement precision of a custom smartphone balance application. Sixty-nine healthy, community-dwelling middle-aged and older adults (ages 41–76 years) performed a 5 s home-based balance assessment weekly for 3 weeks. The assessment was directed by the application, which generated accelerometer-based sway metrics. Participants performed two 30 s trials each of feet together and tandem stance (eyes open, eyes closed) and single leg stance (eyes open only). Intraclass correlation coefficients (ICCs) and relative standard error of measurement (SEM%) were calculated for time-domain and frequency-domain measures. Outcomes achieved good-to-excellent reliability when averaged across three weekly assessments (ICC = 0.59–0.95), particularly mediolaterally. Time-domain measures, particularly mean acceleration (ICC = 0.84–0.92; SEM% = 11.1–23.5%), were the most reliable and precise outcomes overall, while frequency-domain measures showed lower reliability and precision at higher spectral bands (ICC as low as 0.59; SEM% up to 41.5%). As standing tasks became more difficult, reliability and precision declined correspondingly. We conclude that minimally supervised, home-based balance assessment can produce reliable, precise sway measures in healthy, screened, iPhone-owning middle-aged and older adults. This application carries potential for large-scale, remote balance monitoring for research purposes, though validation in more diverse and higher-risk populations will be needed before clinical application can be recommended.

## 1. Introduction

Falls are the leading cause of serious and fatal injuries among older adults [1]. Developing tools to understand balance decline before a first fall is of paramount importance. Clinical tests may not be sufficiently sensitive to detect early decline in balance [2,3] occurring between middle and older age [4,5]. Objective balance tests using force platforms and optic motion capture are expensive and non-portable, restricting testing to laboratory settings. Relying solely on lab testing reduces accessibility to large populations of middle-aged and older adults and limits the feasibility of testing participants repeatedly to register change over time.

The American Association of Retired Persons (AARP) estimates that 91% of adults aged 50 and older own a smartphone [6] and older adults generally report positive attitudes towards technologies designed to support health and physical activity [7]. Smartphones equipped with onboard inertial sensors are capable of approximating center of mass (COM) acceleration during standing balance [8]. Smartphone-derived COM acceleration outcomes are sensitive to visual and task conditions (i.e., eyes open vs. eyes closed, bipedal vs. tandem stance [9]), and discriminate between fallers and non-fallers amongst older adults [8]. In addition, remote balance measurement using participant-owned devices was found to be feasible, safe, and sensitive to differences between young and older adults in standing tasks [10].

Smartphone accelerometry can potentially allow for repeated balance assessment in community settings, yet the reliability of this tool across various tasks and outcome measures must be rigorously established first. Intraclass correlation (ICC) models are widely used to quantify the reliability of test measures, though the type of ICC should be carefully chosen in conjunction with the study’s aims and design [11]. In the context of a weekly balance assessment, the traditional test–retest model ICC(2,1) represents the reliability of a single assessment occasion. Postural sway is known to vary across short-term measurement in both younger and aging adults, due to many non-systematic factors including time of day [12], mental fatigue [13], and arousal [14]. ICC(2,*k*) indicates whether an average of *k* tests is sufficiently reliable to detect longitudinal change and is frequently used in addition to ICC(2,1) when the behavior being measured on a single test is expected to vary in the short-term. It is thus recommended to report both ICC models, understanding that ICC(2,*k*) will always achieve higher values than ICC(2,1) due to the variance reduction produced by averaging tests.

The standard error of measurement (SEM) estimates the relationship between sample variability and sample size on a specific measure. SEM represents the precision of individual test scores in the same units as the test and can provide useful context to ICC outcomes [15]. Normalizing SEM to the measure’s mean (the relative SEM%) allows comparison between multiple outcome measures with different units [16]. Middle-aged and older adults demonstrate lower postural control complexity than younger adults, especially as balance tasks become more challenging, which can result in decreased within- and between-trial variance [17,18]. Counter-intuitively, this reduced variance allows middle-aged and older adults to achieve relatively high ICC values for standing balance tasks [19]. It is thus particularly important to report SEM outcomes in context with ICC measures in these populations [20,21].

While SEM% quantifies measurement precision, it does not indicate how large an observed change must be in order to be considered genuine rather than attributable to measurement error. The minimal detectable change (MDC95) addresses this by establishing with 95% confidence the smallest change in a given outcome that exceeds the bounds of measurement error [15]. Expressed as a percentage of the outcome mean (MDC95%), this metric is particularly relevant to the intended longitudinal application of this tool, as it defines a practical threshold above which a change between assessments can be interpreted as reflecting true change in balance performance rather than noise.

Reliability studies of smartphone-based balance assessment remain limited and heterogeneous in design, and typically used researcher-provided devices administered in a laboratory or clinic rather than participants’ own smartphones used independently at home. Existing work reflects excellent same-day, single-session reliability in a novel application validation [22]; reliability restricted to a minority of outcomes and conditions when the device is strapped to the lower back [23]; and good-to-high between-session reliability when a repeated balance task was administered at 1-week intervals, though testing required in-person researcher support and the device was fixed to an unstable platform rather than mounted on the body [24]. Even applications explicitly motivated by remote or telehealth use have so far been tested in supervised laboratory or clinic sessions using study-issued hardware [25,26]. A detailed comparison of these studies’ devices, populations, settings, tasks, and reliability outcomes is provided in Supplementary Materials (Table S1). To our knowledge, no published study has established the test–retest reliability of a smartphone balance assessment administered longitudinally, unsupervised, on participants’ own devices in their homes, which are the specific conditions under which such a tool would need to perform for real-world longitudinal monitoring.

This study employed a custom iPhone balance assessment application and aimed to (1) evaluate the usability the application when used at home, unsupervised after an initial remote safety and instructions screening, by middle-aged and older adults; (2) assess the single-measure and average-measures test–retest reliability of a comprehensive set of outcomes, including time-domain (reflecting postural steadiness) and frequency-domain measures (reflecting postural strategy); and (3) identify which tasks and measures yield the highest ICCs and the lowest relative SEM% using our protocol.

## 2. Materials and Methods

### 2.1. Participants

Ethical approval for this study was obtained from the New York University Committee on Activities Involving Human Subjects. Eligible participants were healthy adults aged 40 years or older, recruited via word-of-mouth referral, social media outreach, and flyers posted in the community. Individuals were excluded if they self-reported a neurological, sensory, or motor condition; were actively being treated for an orthopedic issue; or were experiencing acute musculoskeletal pain at the time of screening. All participants were required to have an iPhone (Apple Inc., Cupertino, CA, USA) running iOS 12.2 or newer, along with access to videoconferencing software on a home computer or tablet.

We contacted and screened 114 individuals, of whom 99 were assigned a subject ID and began remote testing. Of the 99 who began testing, 69 (70%) completed all three weekly assessments and were included in the final analysis, while 30 did not complete the full protocol (e.g., lost to follow-up, no-show, or discontinued after the first or second session). Of the trials across all completed assessments, 40 trials (3.8%) were missing, most commonly due to a single condition not being completed or uploaded within a given weekly session rather than a participant missing an entire week. A total of 69 healthy adults were included in analyses, ranging from 41–76 years old (see Table 1). When parsed into 10-year age bands (40–49, 50–59, 60–69, 70–79), no group differences were found for average minutes of weekly exercise or perceived intensity of weekly exercise.

Activities-specific Balance Confidence (ABC) Scale scores, collected at baseline, indicated high self-reported balance confidence overall (n = 69; M = 91.2%, SD = 10.5%; range 50–100%), with no significant difference across age bands (F = 1.79, *p* = 0.158).

### 2.2. Procedure

**Figure 1:**
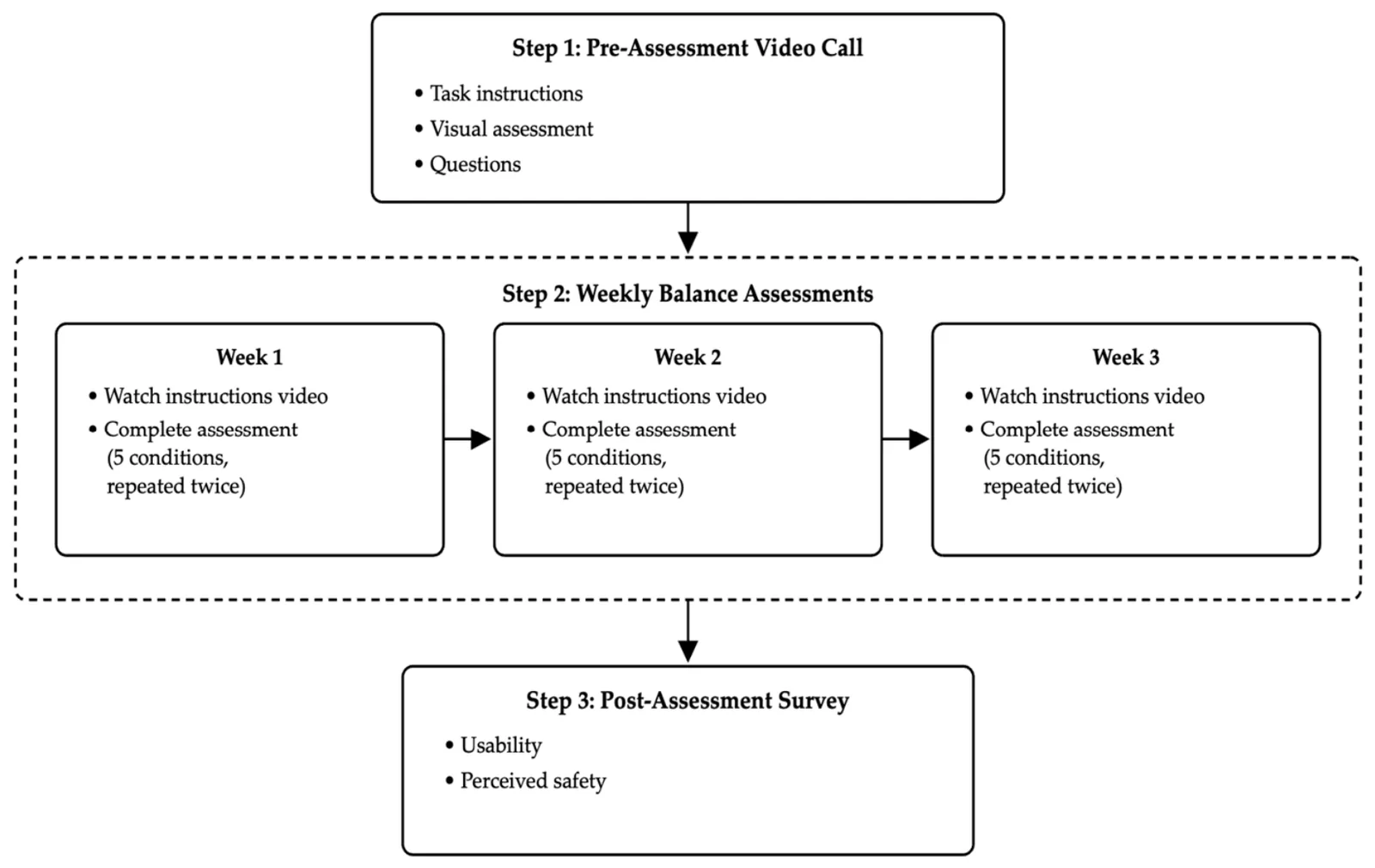
Flow of participant activities.

Each participant met with a member of the research team for a 20 min videoconference (Zoom, Version 6.0; Zoom Video Communications Inc., San Jose, CA, USA [27]) to obtain verbal informed consent to participate in the study activities, to evaluate their ability to safely perform the standing tasks, and to confirm their understanding of the task instructions (see Figure 1 for flow of participant activities). This screening identified participants who were able to stand with feet together, in tandem, and on a single leg unassisted. Any participant over the age of 65 was required to complete the screening with a family member or friend present.

**Figure 2:**
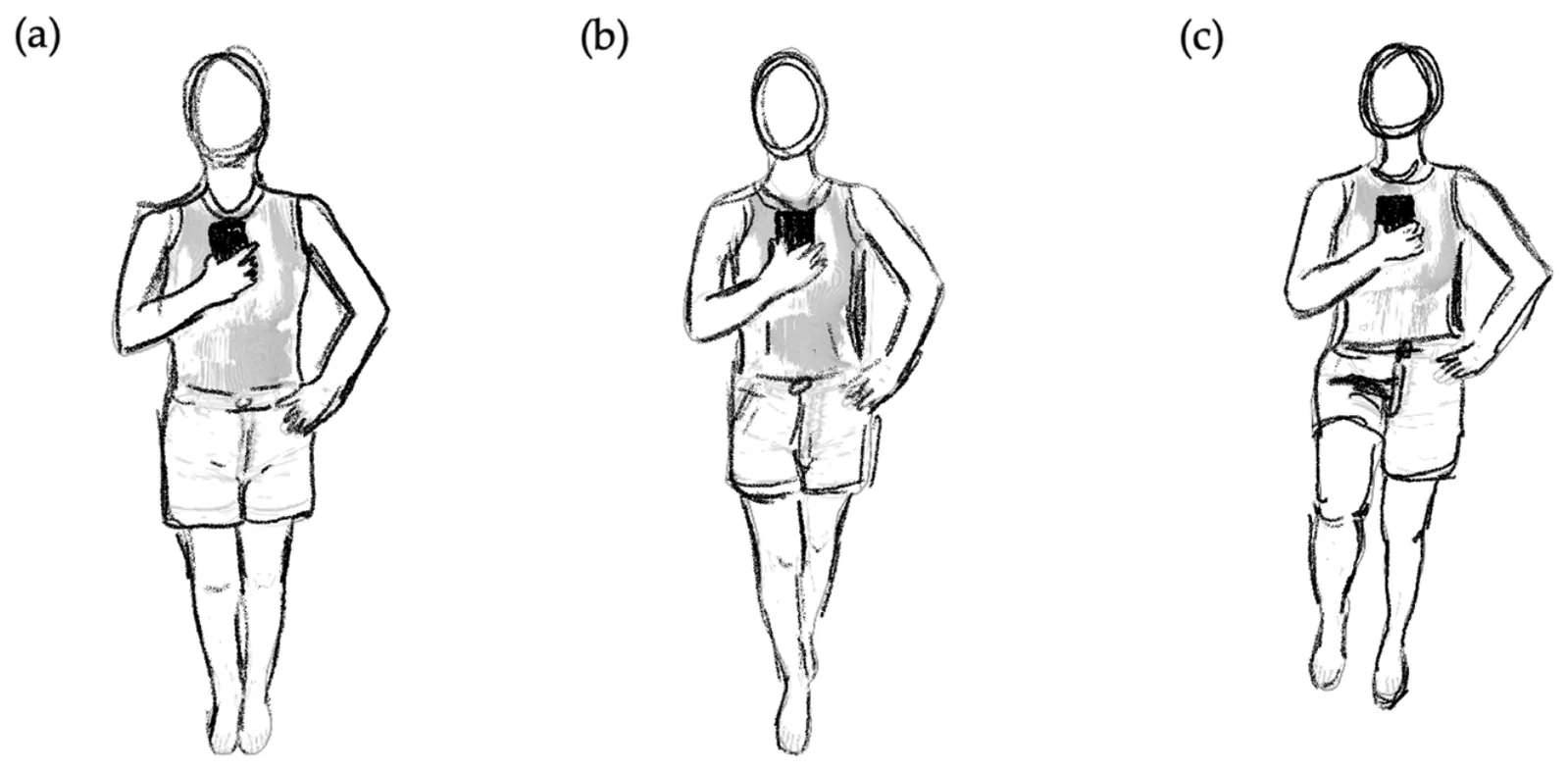
Participants stood (**a**) feet together, (**b**) tandem, and (**c**) single leg.

**Figure 3:**
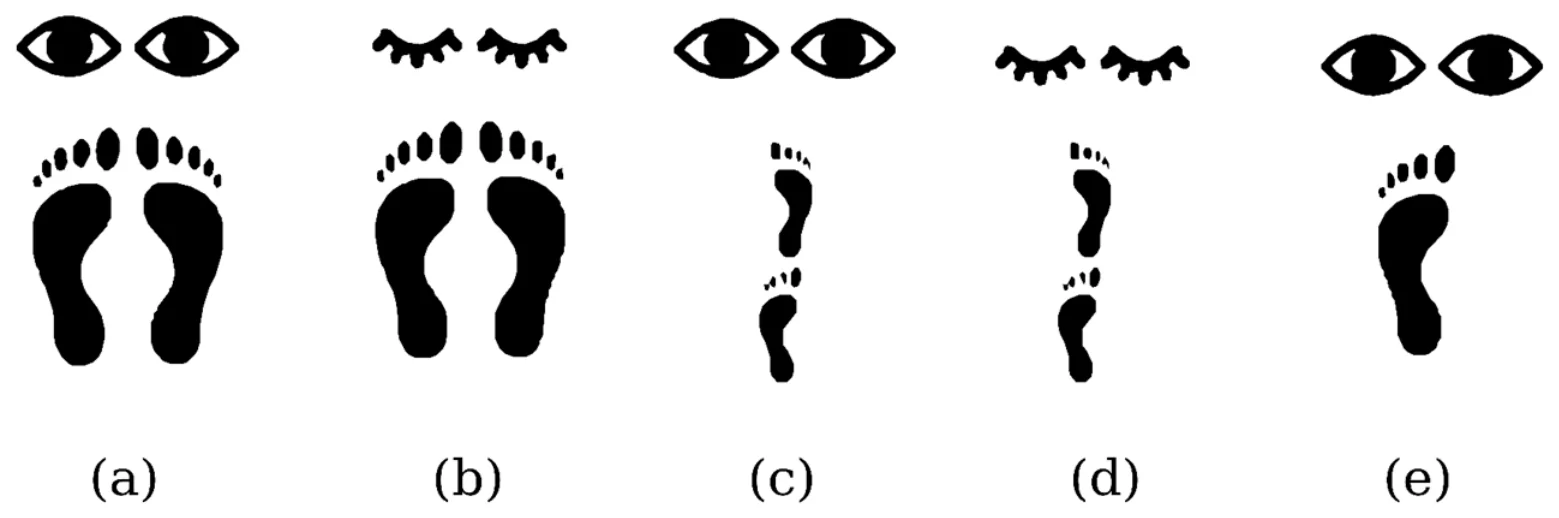
Randomized conditions included (**a**) feet together/eyes open, (**b**) feet together/eyes closed, (**c**) tandem/eyes open, (**d**) tandem/eyes closed, and (**e**) single leg/eyes open.

After completing the initial screening, participants downloaded the application to their phone and watched a short video explaining the procedure; they also completed an online demographic survey, including average weekly physical activity duration (minutes) and intensity (Borg Perceived Exertion scale [28]), and an online version of the Activities-specific Balance Confidence Scale [29]. Over the following three weeks, participants were prompted via customized text messages from the researchers to complete a balance assessment one week after their last assessment at approximately the same time of day. For the remote assessments the application provided auditory instructions for participants to stand barefoot on a non-carpeted surface near a chair or counter which they could use to steady themselves if necessary. Participants were guided during the initial video screening to hold the phone in one hand, screen facing outward, held vertically flush with the center of the chest, with the top of the phone pointing up; the other hand rested on the hip. Phone placement was practiced and verified once during this initial screening call; there was no ongoing app-based or video-based check of phone placement during the subsequent unsupervised weekly assessments. Participants held the phone against clothing rather than skin, and use of a phone case was not monitored or restricted. Holding the phone in this position, participants were guided through a block of randomized 30 s trials: standing quietly with feet together (FT) or in tandem stance (TAN) with eyes open (EO) or closed (EC), and standing on single leg (SL) with eyes open (Figure 2). For TAN stance participants were guided to stand with their dominant leg (“the leg you would kick a ball with”) in front, and for SL stances participants were guided to stand with the dominant leg lifted (Figure 3). Each condition was presented twice, for a total of 10 trials such that the entire procedure took approximately 5 min. Each participant received a newly randomized order of conditions for each weekly assessment and assessments were fully randomized between participants. At the end of each assessment, participants completed a modified System Usability Survey [30] via an online survey.

### 2.3. Experimental Settings

Data were collected using participants’ personal iPhones on which the study application was installed. Devices were not restricted to a single model, consistent with the study’s aim of evaluating the application under real-world conditions on participant-owned hardware rather than a standardized lab device. All iPhone models employed in this study Bosch Sensortec inertial measurement units (IMUs; Bosch Sensortec GmbH, Rutlingen, Germany) as the built-in triaxial accelerometer. However, Apple customizes and integrates these components proprietarily, and exact sensor specifications (e.g., range, resolution, and noise characteristics) are not publicly disclosed by the manufacturer and therefore cannot be reported. Acceleration data were sampled at 100 Hz and streamed in real time to an encrypted database for offline processing.

Testing took place in participants’ home environments; the application instructed participants to stand barefoot on a non-carpeted surface, positioned near a chair or counter for safety. As this was a remote, unsupervised protocol, the testing environment (lighting, ambient space) was not standardized beyond these auditory instructions.

### 2.4. Data Processing

Acceleration was obtained using the iPhone operating system’s native motion-processing framework. This framework combines the raw triaxial accelerometer signal, which reflects both the gravitational component and user-generated motion, with continuous device orientation estimates from the onboard gyroscope to compute the expected gravity vector in the device’s own frame of reference at each sample; this estimated gravity vector is then subtracted from the raw accelerometer signal to yield gravity-compensated acceleration. Gravity-compensated acceleration was output in units of gravitational acceleration (g); the application converted g values to m/s^2^ by multiplying by the standard gravitational constant (9.81 m/s^2^ per g), which was the only calibration step applied to the acceleration signal.

Data were then processed offline using Python (version 3.13.7; Python Software Foundation, Wilmington, DE, USA) [31] to apply a 4th order low-pass Butterworth filter with a cutoff frequency of 10 Hz [32]. The initial 5 s segment of each trial was discarded to exclude transient device motion from screen-tapping or arm positioning at trial onset. Because handheld phone orientation about the sagittal axis was not identical across participants or sessions, mediolateral body acceleration was derived from the vector resultant of the device’s horizontal and vertical acceleration channels, rather than from the horizontal channel alone. There is negligible vertical excursion of the trunk during quiet standing [10,33], meaning the resultant magnitude closely tracks true mediolateral sway independent of how the phone happened to be rotated when held against the chest. We previously employed the same coordinate transformation used in our prior validation of this application [10], wherein all participants were remotely monitored in real time by research staff while performing the same tasks as presented here.

Trial-level outliers were identified using a 3 standard deviation (3SD) threshold; trials with values exceeding 3SD from the sample mean for a given outcome were considered outliers and excluded. Approximately 4% of all trials were removed on this basis, and these excluded trials were evenly distributed across subjects rather than concentrated in a small number of participants. All remaining trials were included in the analyses reported here.

### 2.5. Outcome Measures and Statistical Analyses

Outcomes derived from anterior–posterior (AP) and ML trunk acceleration at 100 Hz included: mean absolute acceleration (Mean Accel), root mean square acceleration (RMSA), total power spectral density (PSD), and PSD in four bands: PSD1: <0.25 Hz, PSD2:0.25–0.5 Hz, PSD3: 0.5–2 Hz, and PSD4: 2–10 Hz.

Power spectral density was estimated for each 30 s trial, following removal of the first 5 s as described above (25 s of data per trial, 2500 samples at 100 Hz). For each direction (AP, ML) we employed Welch’s method with a Hanning window, an FFT segment length of 1024 samples (10.24 s), and no overlap between segments. This yielded a frequency resolution of 0.098 Hz (100 Hz/1024 samples). No detrending was applied prior to spectral estimation. The resulting one-sided spectrum was scaled to power per unit frequency ((m/s^2^)^2^/Hz). Power was integrated using the trapezoidal rule over each of the five frequency bands (total and PSD1–PSD4). The distribution of spectral power appears to change with age [34,35], with older adults demonstrating higher total power than younger adults, decreased power in lower frequency bands and increased power in higher frequency bands [34]. Decreased power in low frequency bands has been associated with increased fall risk in older adults [35], suggesting the importance of measuring both time-domain and frequency-domain outcomes in this population.

Each assessment included two trials per condition, the outcomes of which were averaged prior to ICC calculation. Test–retest reliability was calculated using two-way random-effects ICCs with absolute agreement, treating test day as a random factor to allow generalization of reliability estimates beyond the three specific assessment occasions, for both a single assessment, ICC(2,1), and the average of three assessments, ICC(2,3) [11], computed using the Pingouin package (Version 0.6.1; https://pingouin-stats.org; accessed 15 August 2026) in Python [36]. Reliability was interpreted using the following cutoffs: <0.5: poor, 0.5–0.75: moderate, 0.75–0.9: good, >0.9: excellent [37]. No a priori sample size calculation was performed for this study. Recruitment began as a pilot with an initial target of approximately 12 participants intended to generate preliminary estimates for a future, formally powered study; enrollment continued beyond this original target as participant interest exceeded expectations, yielding the final analytic sample reported here. In place of an a priori power calculation, the precision actually achieved with this sample is reflected directly in the 95% confidence intervals reported for each ICC estimate throughout the Results.

SEM% was derived from the standard deviation and corresponding ICC for each model, expressed as a percentage of the mean so that error could be compared across outcomes with different units:

$$SEM\%\;=\;(SD\times \surd (1-ICC))/\bar{x}\;\times \;100$$

To contextualize measurement precision in terms of detectable change, the minimal detectable change at the 95% confidence level (MDC95%) was calculated for each outcome and expressed relative to the outcome mean [15]:

MDC95% = 1.96 × √2 × SEM%

MDC95% was calculated using SEM% derived from both ICC(2,1) and ICC(2,3), reflecting the change required to exceed measurement error using a single assessment versus the average of three assessments, respectively.

Unlike ICC, Relative SEM% and MDC95% do not have a widely accepted qualitative classification system (e.g., poor, moderate, good, excellent); literature instead recommends interpreting a given SEM% or MDC95% value relative to the specific analytical or clinical goals of the measurement application, rather than against a fixed universal threshold [38,39]. We therefore report these metrics primarily to enable comparison of relative precision across our own outcome measures and tasks.

Finally, a matrix comparing ICC and SEM% outcomes by direction (AP, ML) and task (FT/EO, FT/EC, TAN/EO, TAN/EC, SL/EO) was created to indicate the individual outcome measures that achieved highest ICC and lowest SEM% simultaneously. All signal processing and statistical analyses were performed in Python [31], using SciPy (version 1.17.0; https://scipy.org, accessed 15 August 2026) [40] for filtering and spectral analysis and the Pingouin statistics package [36] for reliability analyses.

Claude Sonnet 4.6 (Anthropic, San Francisco, CA, USA) [41] was used to assist in the preparation and formatting of figures. The underlying data, analyses, and final figure content were generated, reviewed, and approved by the authors.

The de-identified, processed data supporting the conclusions of this article are openly available in a public repository at https://zenodo.org/records/21499321 (accessed on 22 July 2026).

## 3. Results

### 3.1. Usability and Protocol Adherence

Modified System Usability Survey (SUS) responses, examined among the 69 participants who completed all three weekly assessments, showed consistently high usability and protocol adherence across the three-week protocol (composite M = 4.61–4.62 out of 5 each week; Table 2), with minimal week-to-week change on any individual item (Δ ≤ 0.11 points). No falls, near falls, or adverse events were reported.

### 3.2. Test–Retest Reliability of Anterior–Posterior Outcomes

**Figure 4:**
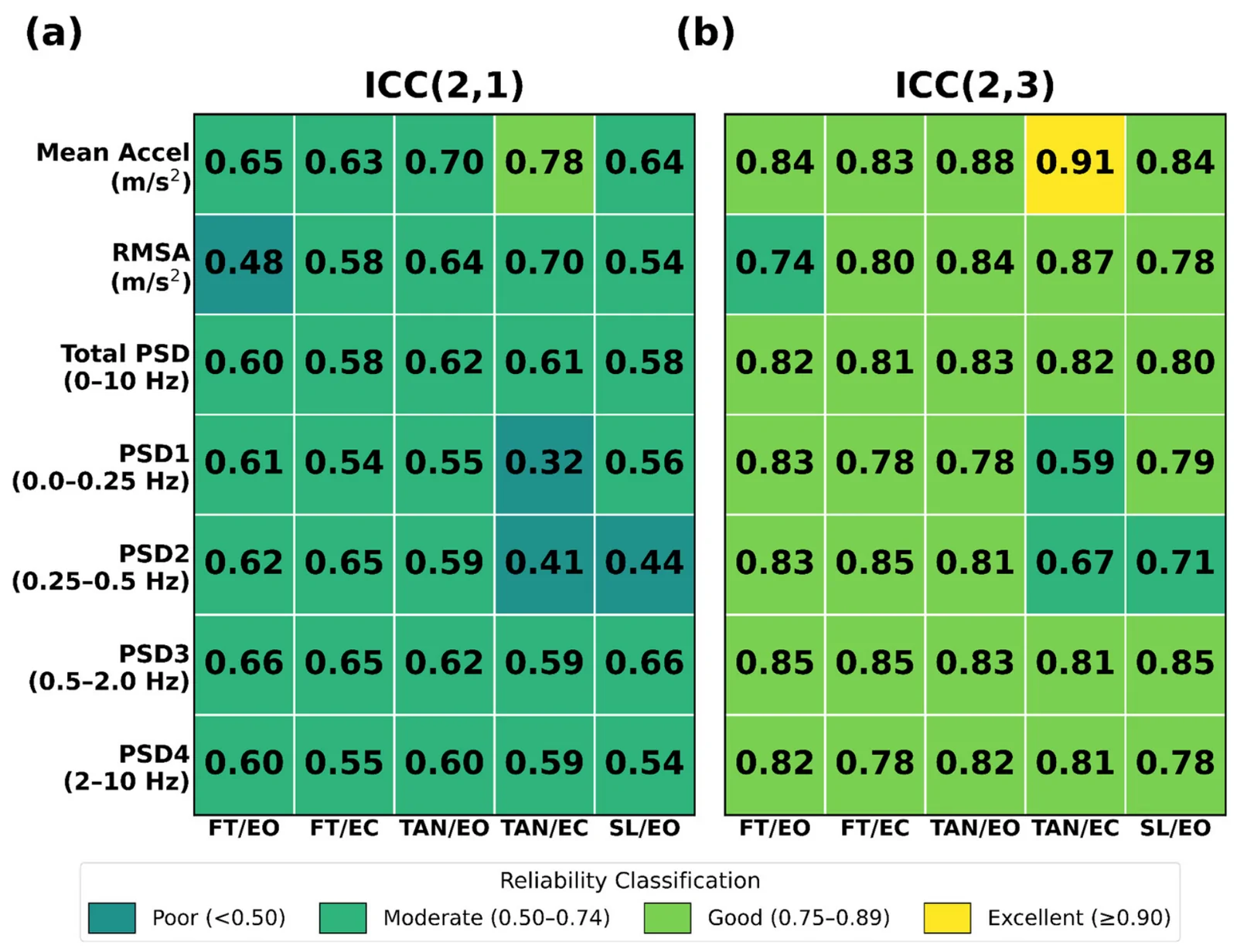
(**a**) Anterior–posterior single-assessment intraclass correlation coefficients by task and measure. (**b**) Anterior-posterior averaged-assessment intraclass correlation coefficients by task and measure. ICC: intraclass correlation coefficient; Mean Accel: mean acceleration; RMSA: root mean square acceleration; PSD: power spectral density; FT: feet together; TAN: tandem; SL: single leg; EO: eyes open; EC: eyes closed.

Intraclass correlation coefficients for AP outcomes are presented for both single-measure (ICC(2,1)) and average-measure (ICC(2,3)) estimates across all stance conditions (see Figure 4). All ICC and SEM% outcomes along with their 95% confidence intervals by direction, task, and condition may be found in Table S2 in Supplementary Materials. Representative traces depicting trial-level absolute value acceleration and PSD may be found in Figures S1 and S2, respectively. ICC(2,1) values ranged from poor to good reliability (ICC = 0.32–0.78), with the majority of measures falling within the moderate range (0.50–0.74) and only one value, Mean Accel during tandem/eyes closed (ICC = 0.78 [0.68, 0.85]), in the good range. The lowest reliability was observed for PSD1 and PSD2 during the tandem stance with eyes closed condition (TAN/EC; ICC = 0.32 [95% CI: 0.17, 0.48] and 0.41 [0.25, 0.56], respectively). Mean Accel demonstrated moderate to good reliability across conditions (ICC = 0.63–0.78), with the highest single-measure ICC observed during tandem/eyes closed (ICC = 0.78 [0.68, 0.85]).

ICC(2,3) values ranged from moderate to excellent reliability (ICC = 0.59–0.91). The majority of ICC(2,3) values fell within the good range (0.75–0.89), with only one value classified as excellent: Mean Accel during tandem/eyes closed (ICC = 0.91 [0.87, 0.94]). Mean Accel during feet together/eyes open (ICC = 0.84 [0.77, 0.90]) fell within the good range. PSD1 during tandem/eyes closed demonstrated the lowest average-measure reliability (ICC = 0.59 [0.38, 0.74]).

### 3.3. Relative Standard Error of Measurement and Minimal Detectable Change of Anterior–Posterior Outcomes

**Figure 5:**
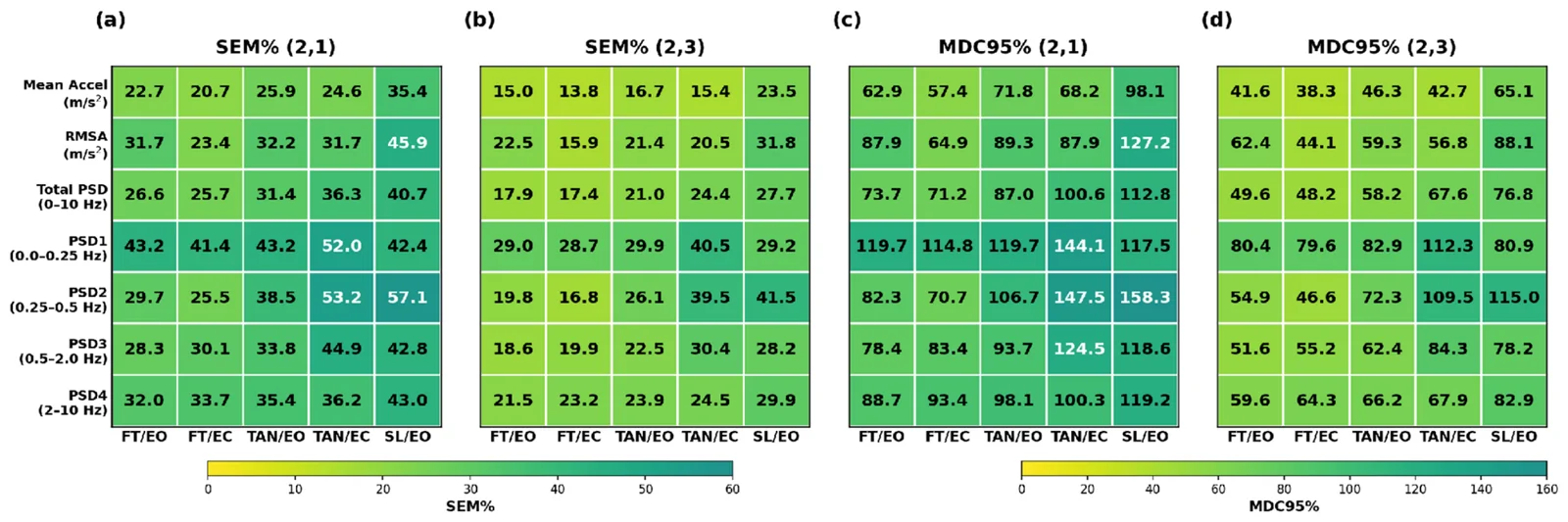
Anterior–posterior relative standard error of measurement and minimal detectable change by task and measure. (**a**) SEM%(2,1); (**b**) SEM%(2,3); (**c**) MDC95%(2,1); (**d**) MDC95%(2,3). SEM%: standard error of measurement; MDC95%: minimal detectable change at the 95% confidence level; Mean Accel: mean acceleration; RMSA: root mean square acceleration; PSD: power spectral density; FT: feet together; TAN: tandem; SL: single leg; EO: eyes open; EC: eyes closed.

Relative SEM (SEM%) for AP acceleration measures are presented for both single-measure (ICC(2,1)) and average-measure (ICC(2,3)) estimates across all stance conditions (see Figure 5). ICC(2,1) SEM% values ranged from 20.7% to 57.1%, with the lowest measurement error observed for Mean Accel during feet together/eyes closed (20.7%) and the highest for PSD2 during single leg/eyes open (57.1%).

ICC(2,3) SEM% values ranged from 13.8% to 41.5%. Mean Accel demonstrated the lowest average-measure SEM% values across conditions (13.8–23.5%), with the best precision achieved during feet together/eyes closed (13.8%) and FT/EO (15.0%).

MDC95% mirrored this pattern, ranging from 38.3% to 158.3% across conditions. Mean Accel required the smallest relative change to exceed measurement error, from 38.3% (FT/EC) to 65.1% (SL/EO), while PSD1 and PSD2 required considerably larger changes, exceeding 100% in the more difficult tandem/eyes closed and single leg conditions (see Figure 5c,d).

### 3.4. Test–Retest Reliability of Mediolateral Outcomes

**Figure 6:**
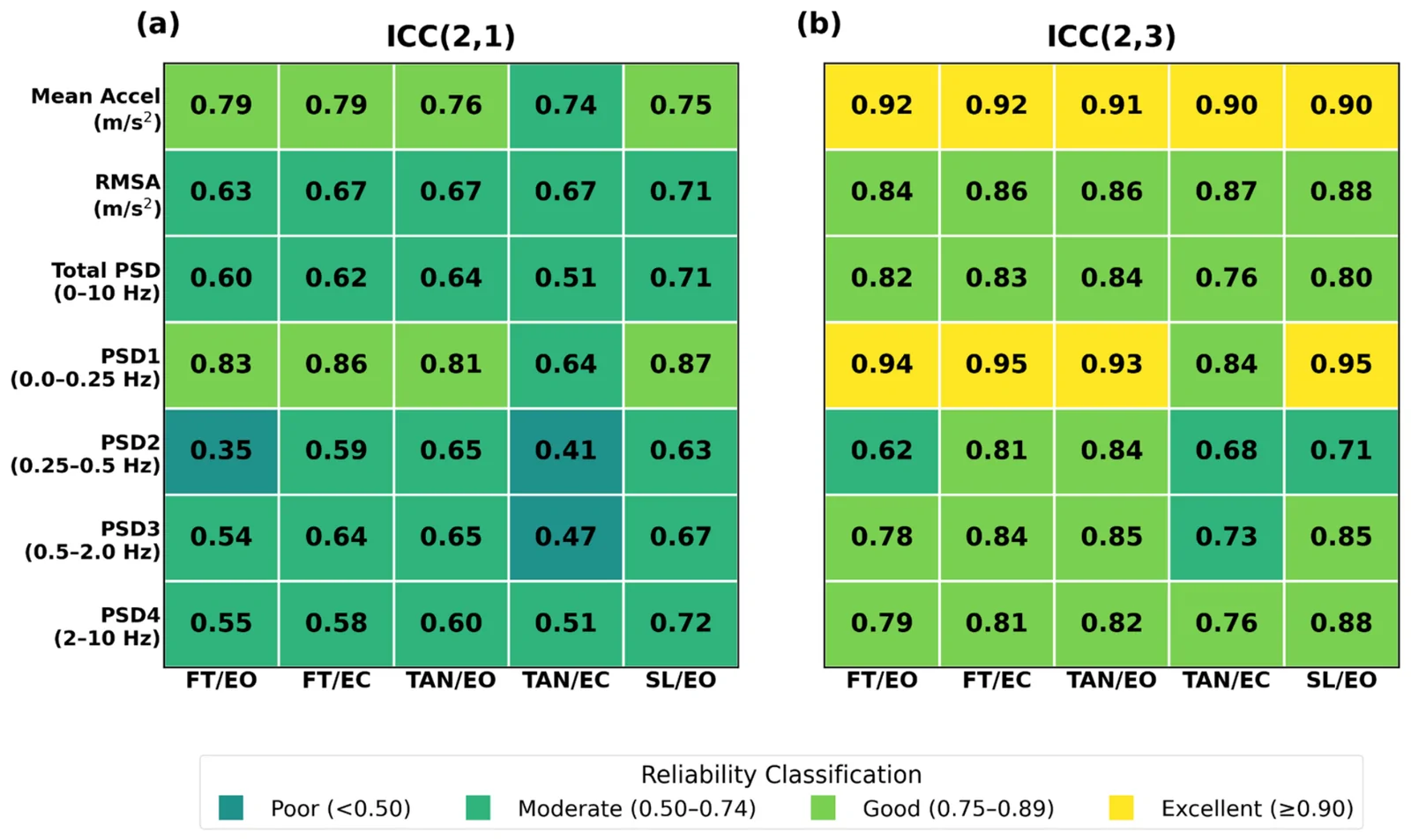
(**a**) Mediolateral single-assessment intraclass correlation coefficients by task and measure. (**b**) Mediolateral averaged-assessment intraclass correlation coefficients by task and measure. ICC: intraclass correlation coefficient; Mean Accel: mean acceleration; RMSA: root mean square acceleration; PSD: power spectral density; FT: feet together; TAN: tandem; SL: single leg; EO: eyes open; EC: eyes closed.

Intraclass correlation coefficients for ML acceleration measures are presented for both single-measure (ICC(2,1)) and average-measure (ICC(2,3)) estimates across all stance conditions (see Figure 6). ICC(2,1) values ranged from poor to good reliability (ICC = 0.35–0.87). The lowest single-measure reliability was observed for PSD2 during feet together/eyes open (ICC = 0.35 [95% CI: 0.19, 0.51]) and PSD3 during tandem/eyes closed (ICC = 0.47 [0.32, 0.61]). PSD1 demonstrated consistently good single-measure reliability across most conditions (ICC = 0.81–0.87), with the exception of tandem/eyes closed (ICC = 0.64 [0.51, 0.75]). Mean Accel also showed moderate to good ICC(2,1) reliability across all conditions (ICC = 0.74–0.79).

ICC(2,3) values ranged from moderate to excellent reliability (ICC = 0.62–0.95). PSD1 demonstrated good to excellent average-measure reliability across all conditions (ICC = 0.84–0.95), with the highest values observed for feet together/eyes open (ICC = 0.94 [0.90, 0.96]), feet together/eyes closed (ICC = 0.95 [0.92, 0.97]), and single leg/eyes open (ICC = 0.95 [0.93, 0.97]). Mean Accel also achieved excellent ICC(2,3) values across all five conditions (ICC = 0.90–0.92). The lowest average-measure reliability was observed for PSD2 during feet together/eyes open (ICC = 0.62 [0.42, 0.76]), one of four ICC(2,3) values falling below the good threshold, along with PSD2 during tandem/eyes closed (0.68), PSD2 during single leg/eyes open (0.71), and PSD3 during tandem/eyes closed (0.73).

### 3.5. Relative Standard Error of Measurement and Minimal Detectable Change of Mediolateral Outcomes

**Figure 7:**
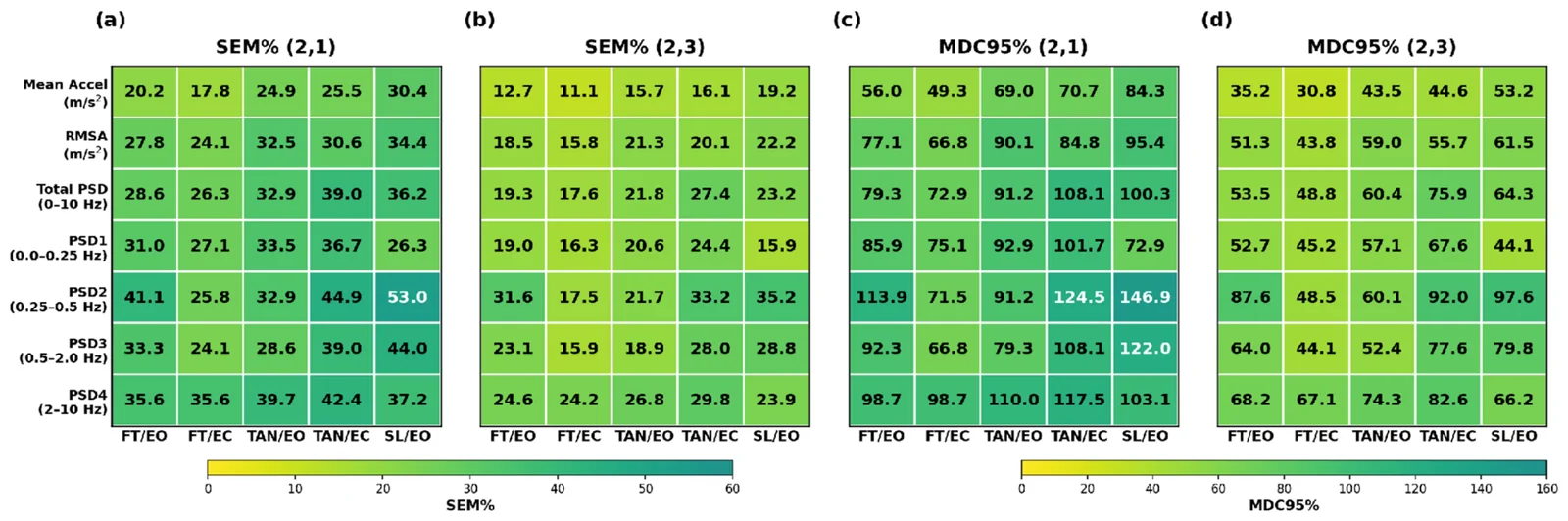
Mediolateral relative standard error of measurement and minimal detectable change by task and measure. (**a**) SEM%(2,1); (**b**) SEM%(2,3); (**c**) MDC95%(2,1); (**d**) MDC95%(2,3). SEM%: standard error of measurement; MDC95%: minimal detectable change at the 95% confidence level; Mean Accel: mean acceleration; RMSA: root mean square acceleration; PSD: power spectral density; FT: feet together; TAN: tandem; SL: single leg; EO: eyes open; EC: eyes closed.

Relative standard error of measurement (SEM%) for ML acceleration measures are presented for both single-measure (ICC(2,1)) and average-measure (ICC(2,3)) estimates across all stance conditions (see Figure 7). ICC(2,1) SEM% values ranged from 17.8% to 53.0%, with the lowest measurement error observed for Mean Accel during feet together/eyes closed (17.8%) and the highest for PSD2 during single leg/eyes open (53.0%).

ICC(2,3) SEM% values ranged from 11.1% to 35.2%. Mean Accel demonstrated the lowest average-measure SEM% values (11.1–19.2%), with highest precision achieved during feet together/eyes closed (11.1%) and feet together/eyes open (12.7%). PSD2 remained the least precise measure even with trial averaging, with SEM% values of 31.6% and 35.2% under feet together/eyes open and single leg/eyes open conditions, respectively.

MDC95% values in the ML direction were consistently lower than AP, ranging from 30.8% to 115.0%. Mean Accel again required the smallest relative change to exceed measurement error (30.8–53.2%), reinforcing its advantage as a monitoring outcome, while PSD2 required changes exceeding 90% in the more difficult conditions (see Figure 7c,d).

### 3.6. Combined Reliability and Precision

**Figure 8:**
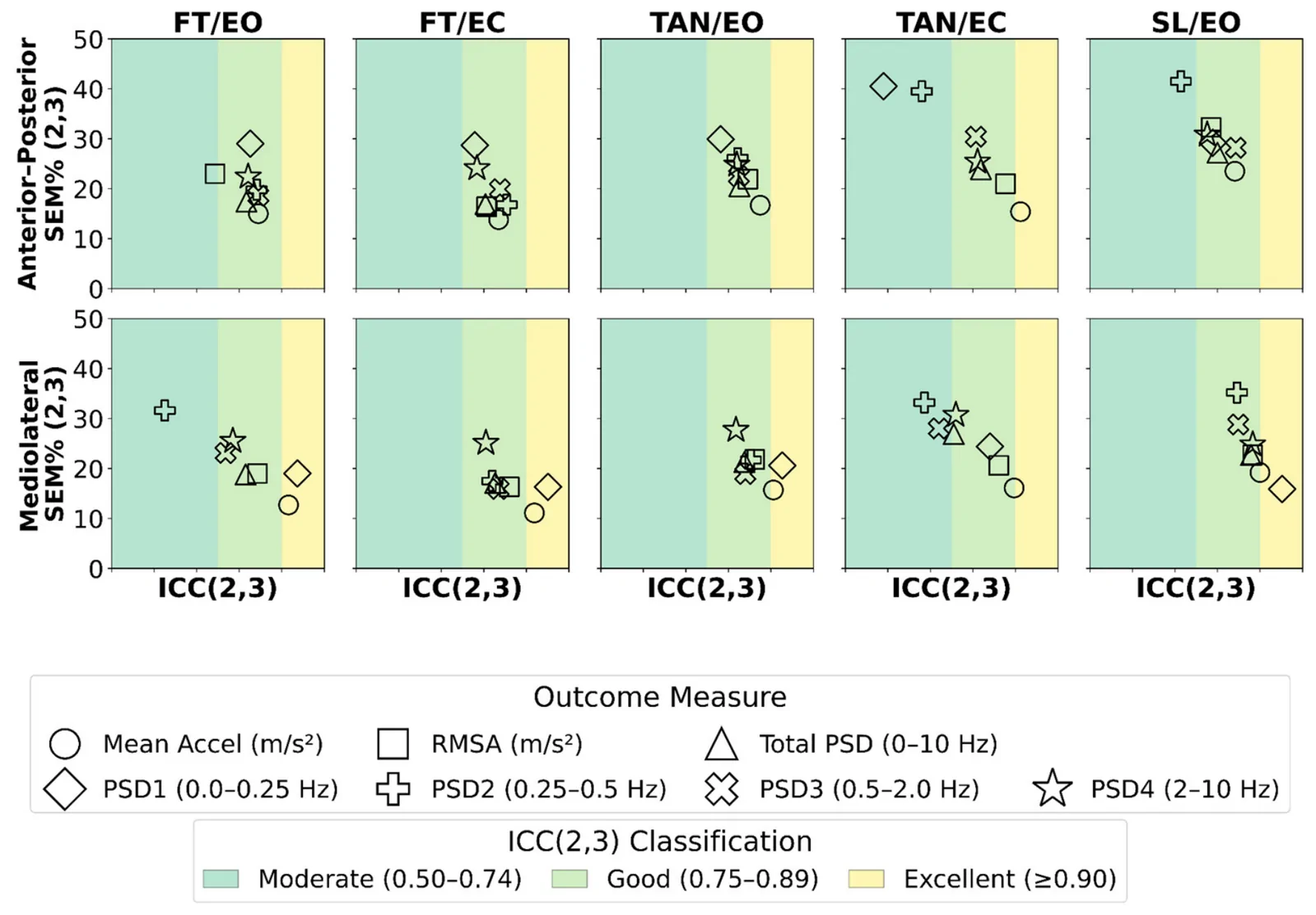
Combined reliability and precision by task. SEM%: standard error of measurement; ICC: intraclass correlation coefficient; Mean Accel: mean acceleration; RMSA: root mean square acceleration; PSD: power spectral density; FT: feet together; TAN: tandem; SL: single leg; EO: eyes open; EC: eyes closed.

**Figure 9:**
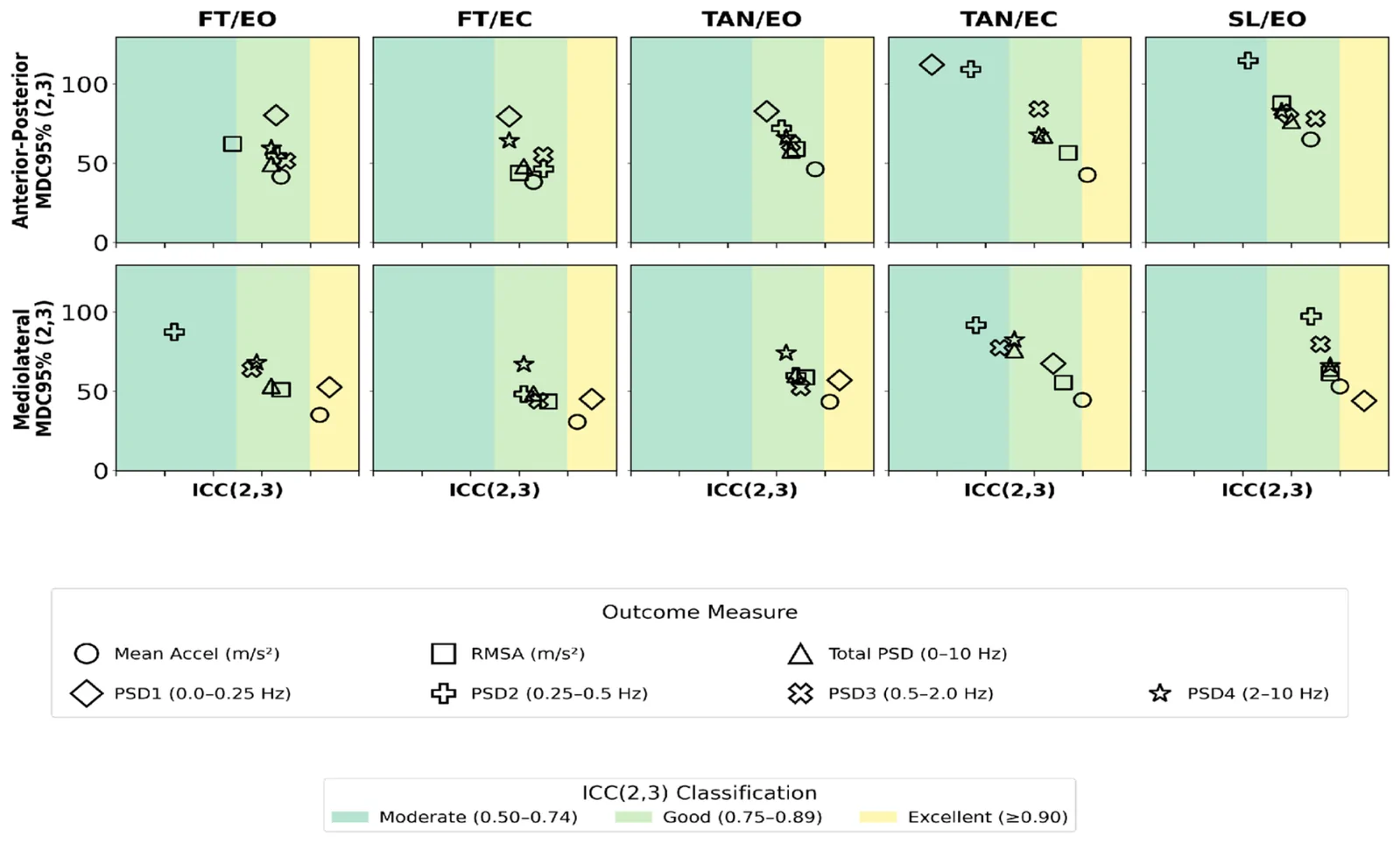
Comparison of minimal detectable change (MDC95%) and intraclass correlation coefficient (ICC(2,3)) by task, direction, and outcome measure. Background shading indicates ICC(2,3) classification: moderate (0.50–0.74), good (0.75–0.89), excellent (≥0.90). MDC95%: minimal detectable change at the 95% confidence level; ICC: intraclass correlation coefficient; Mean Accel: mean acceleration; RMSA: root mean square acceleration; PSD: power spectral density; FT: feet together; TAN: tandem; SL: single leg; EO: eyes open; EC: eyes closed.

Across all outcome measures and stance conditions evaluated, Mean Accel in the mediolateral direction (averaged across three assessments) demonstrated the most consistent reliability (ICC(2,3) = 0.90–0.92) and lowest measurement error (SEM% = 11.1–19.2%), indicating congruence between reliability and precision for this measure (see Figure 8 and Figure 9). In contrast, several PSD-band measures (particularly PSD2 and PSD4) showed incongruence between the two metrics, with comparatively higher SEM% despite moderate-to-good ICC values, most evident in the more difficult tasks (tandem/eyes closed, single leg/eyes open).

Mediolateral Mean Accel also required the smallest relative change of any outcome to be distinguished from measurement error (30.8–53.2%), whereas PSD-band measures with incongruent ICC and SEM% required correspondingly larger changes. Averaging three assessments reduced the required measurement error by approximately one-third relative to a single assessment across all outcomes, underscoring the practical benefit of repeated testing for longitudinal monitoring.

## 4. Discussion

The purpose of this study was to (1) evaluate the usability of a home-based balance assessment for middle-aged and older adults; (2) assess the single-measure and average-measures test–retest reliability of time-domain and frequency-domain measures; and (3) identify which tasks and measures yield the highest ICCs and the lowest relative SEM% using our protocol. Importantly, after a preliminary video screening, testing was completed fully remotely and unsupervised in participant’s homes, providing real-world insight into how large-scale, longitudinal balance assessments could be deployed in the near future.

We found that by averaging outcomes from three weekly assessments, most outcomes achieved good-to-excellent reliability, a marked improvement over single-assessment ICCs. These findings are consistent with literature employing multiple test averages from forceplate-derived center of pressure (COP) measures [42]. In accordance with these findings and the known day-to-day variability inherent to balance, it appears advisable to average weekly testing outcomes for month-to-month comparison that would indicate age-related changes over time. This discussion will henceforth focus on findings from the multiple test averaged ICC(2,3) and SEM%(2,3).

### 4.1. Usability and Protocol Adherence

On average, participants reported that the application was easy to use and they felt safe while performing the tasks at home. Participants reported that they believed they performed the tasks correctly most or all of the time. The stability of modified SUS scores across the three weekly assessments, with no meaningful week-to-week change in perceived ease of use, felt safety, or protocol adherence, suggests that usability and compliance with unsupervised testing did not degrade with repeated exposure, supporting the feasibility of extending this assessment beyond three weeks for longitudinal monitoring.

### 4.2. Mediolateral Sway Demonstrates Greater Reliability and Precision than Anterior–Posterior Sway

ML reliability was consistently higher than AP across nearly all measures and conditions, with ML frequently reaching excellent (≥0.90) for Mean Accel and PSD1, while only AP Mean Accel for the TAN/EC stance reached ICC(2,3) of 0.91. Relative SEM% followed a behaviorally congruent inverse pattern, with higher measurement error for AP compared to ML, especially for PSD1 and PSD2, where AP values reached above 30% in several conditions. Taken together, using this smartphone sensor balance assessment, ML sway demonstrated both greater reliability and measurement precision than AP sway. This is consistent with sway reliability literature [42,43], and potentially indicative of the narrower stances employed in this study (e.g., feet together, tandem, single leg) wherein ML is the dominant direction of sway. Previously, smartphone sensor-derived ML Mean Accel was found to distinguish between younger and older adults, particularly in tandem stance [10], suggesting these outcomes are not only reliable and precise, but also valid.

### 4.3. ICC and SEM% Vary by Task Difficulty

The findings of this study indicate that as the standing task increases in difficulty on a continuum of FT/EO to SL/EO, ICC(2,3) declines while SEM% rises correspondingly. Previous research highlights the importance of selecting tasks that pose an appropriate challenge for the population, minimizing ceiling effects for younger adults and floor effects for older users [10]. In the context of home-based assessment, participant safety must also be considered, particularly when tasks are performed without direct supervision. Notably, instrumented measures of standing balance substantially enhance the resolution of balance assessment, enabling the detection of meaningful performance differences even during relatively simple postural tasks. The findings of the current study further justify the selection of standing tasks that aging populations can likely perform safely when unsupervised at home and that will provide reliable and precise outcomes. MDC95% outcomes also demonstrated that substantially larger changes are required to exceed measurement error as task difficulty increases. MDC95% offers further context to the cost of task difficulty than ICC or SEM% alone, as it expresses this cost directly in terms of the change a clinician or researcher would need to observe to be confident that true change had occurred.

### 4.4. Time-Domain Outcomes Are More Reliable and Precise than Frequency-Domain Outcomes

Traditional time-domain measures of postural sway (Mean Accel, RMSA) outperformed frequency-domain measures on both reliability and precision, in both AP and ML planes. Mean Accel was the strongest measure overall, with good-to-excellent ICCs (0.84–0.92) and the lowest SEM% (11–24%) across nearly all conditions. As COM Mean Accel (which we approximated using a chest-bound sensor), is biomechanically linked to COP mean velocity, it is not surprising that our findings are consistent with postural sway literature reporting forceplate-derived mean velocity as highly reliable and precise [18,44]. Consistent with these findings, Mean Accel also required the smallest relative change to be distinguished from measurement error, while several higher-frequency PSD bands required changes exceeding 100% of the outcome mean in the more difficult conditions, further supporting Mean Accel as the preferred outcome for detecting longitudinal change with this application.

Among frequency-domain measures, Total PSD (0–10 Hz) and PSD1 (0–0.25 Hz) perform comparably to the traditional time-domain measures, reaching excellent ICCs in ML direction. Conversely, middle-to-higher band PSDs (PSD2–PSD4: spanning 0.25–10 Hz) consistently demonstrated the lowest ICCs (dropping to moderate, 0.59–0.68, in several tasks), and the highest SEM% (often above 30%). This may indicate that reliability and precision degrade as sway moves to higher-frequency bands in middle-aged and older populations, which could be attributable to imitations of the measurement device itself and/or increasing variability inherent in the behavior. However, PSD estimates for this study were derived from a relatively short 25 s effective window (following removal of the initial 5 s) using Welch’s method with a 1024-sample (10.24 s) segment length and no overlap between segments, yielding only approximately two segments available for spectral averaging. Because PSD1 is defined by only two non-zero frequency bins at this resolution (0.098 Hz spacing), its power estimate rests on limited spectral averaging and may carry higher estimate variance than the broader higher-frequency bands. While PSD1 nonetheless demonstrated good-to-excellent reliability in the present sample, longer recording durations or overlapping-segment approaches could improve the stability of low-frequency spectral estimates in future work.

Postural sway literature shows spectral power distribution shifts with age: older adults exhibit higher total power, with power shifting from lower to higher frequency bands [34,35]. Since reduced low-frequency power has been linked to increased fall risk [35], both time- and frequency-domain outcomes are important to measure in this population. Our findings are also broadly consistent with the rambling–trembling hypothesis of postural control [45], which decomposes postural sway into lower (rambling) frequency behaviors associated with longer neural loop/supraspinal/central processing during static sway and higher (trembling) frequency behaviors associated with shorter neural loop/reflexive responses. Supporting our findings, it has been established that especially in middle- and older-age, lower PSD bands are likely inherently more stable than higher bands due to the stochastic nature of trembling parameters [46].

### 4.5. Limitations

The findings of this study may be constrained by several limitations. Firstly, this study evaluated test–retest reliability and measurement precision, not criterion, construct, or predictive validity. Previously, we found that our application distinguished between healthy younger and healthy older adults in the same standing tasks as used here [10], though no reference device (e.g., force platform) or clinical comparator (e.g., established balance measures, fall history, prospective fall outcomes) was incorporated. Therefore, claims regarding remote assessment and clinical application should be read as motivating future work rather than as conclusions supported by the present data. Validating the tool for clinical use will require dedicated studies matched to the intended application. Similarly, while MDC95% provides a statistical threshold for distinguishing true change from measurement error, it does not establish whether a change of this magnitude is clinically meaningful, as the clinical significance of change in these sway metrics has not yet been established.

This study was conducted exclusively on iOS devices, as our application’s accelerometer data collection pipeline is implemented natively for iOS and executes identically regardless of iOS version. Findings may not generalize to Android devices, which involve a more diverse range of hardware and sensor fusion implementations across manufacturers, and would require separate investigation.

The sample was healthy, community-dwelling, and self-selected: participants were required to own or have access to an iPhone, have videoconferencing capability, and be able to perform feet-together, tandem, and single-leg stance unassisted. The sex distribution was also imbalanced, with women comprising 100% of the 70–79 age band. Dedicated validation in more diverse and higher-risk samples will be necessary before this tool’s use for clinical application or large-scale deployment can be recommended.

Finally, the application did not automatically detect trial failure (e.g., stepping, touching the chair or counter, opening the eyes during eyes-closed conditions, losing single-leg stance, or discontinuing a trial partway through); adherence to task instructions was instead assessed via participant self-report on the modified System Usability Survey, which asked participants whether they completed tasks as instructed, maintained eyes closed for the full trial duration, and kept their free hand on their hip throughout. While self-reported adherence was high across all three weeks, self-report cannot substitute for objective, automated verification of trial validity, and some undetected deviations from protocol may have gone unreported.

## 5. Conclusions

These findings support the reliability of selected sway metrics, particularly mediolateral mean acceleration, for smartphone-based, minimally supervised balance assessment in healthy, middle-aged and older adults. Extending this approach to less active, higher-fall-risk, or more diverse populations will require dedicated validation in those groups. Mean acceleration, particularly in the mediolateral direction, demonstrated reliable performance across both relative and absolute reliability metrics, supporting its use as a primary outcome measure for this assessment. In contrast, middle-to-higher frequency power (PSD2-PSD4) exhibited less agreement between relative and absolute reliability indices, indicating that measures with acceptable ICC values may nevertheless be associated with substantial measurement error. These findings suggest that caution is warranted when interpreting higher-frequency spectral metrics, as the observed variability may reflect a combination of measurement limitations and genuine fluctuations in postural control.

## Figures and Tables

**Table 1:** Participant characteristics by age band.

Age Band	40–49	50–59	60–69	70–79	F ^1^	*p*
	n = 9	n = 29	n = 17	n = 14		
**Demographics**						
Age (years)	44.4 (2.7)	54.2 (3.3)	63.9 (3.2)	72.7 (2.2)	—	—
Female, n (%)	7 (77.8)	23 (79.3)	14 (82.4)	14 (100.0)	—	—
Male, n (%)	2 (22.2)	6 (20.7)	3 (17.6)	0 (0.0)	—	—
**Physical Activity**						
Exercise (min/week)	240.6 (90.3)	303.0 (214.6)	229.1 (274.5)	274.6 (292.6)	0.387	0.763
Average Borg Intensity	12.3 (2.1)	12.4 (0.9)	11.5 (3.5)	11.5 (2.0)	0.889	0.453
ABC Scale (%)	94.7 (5.8)	92.5 (10.4)	92.0 (6.6)	85.7 (14.7)	1.789	0.158

^1^ F and *p* values indicate results of one-way ANOVA across age bands (α = 0.05) indicating no significant differences in weekly exercise minutes or intensity.

**Table 2:** Modified System Usability Survey (SUS) item and composite scores by week, among the 69 participants who completed all three weekly assessments. Items scored on a 1–5 scale (1 = strongly disagree/never; 5 = strongly agree/every time).

Item	Week 1 M (SD)	Week 2 M (SD)	Week 3 M (SD)
Ease of use	4.72 (0.56)	4.69 (0.61)	4.78 (0.46)
Task adherence	4.48 (0.84)	4.57 (0.72)	4.46 (0.72)
Eyes-closed compliance	4.74 (0.68)	4.72 (0.68)	4.65 (0.73)
Hand-on-hip compliance	4.35 (0.68)	4.30 (0.77)	4.37 (0.68)
Felt safe	4.80 (0.56)	4.80 (0.53)	4.81 (0.52)
Composite (mean of 5 items)	4.62 (0.49)	4.61 (0.47)	4.61 (0.49)

M = mean; SD = standard deviation. Composite = mean of the five item scores.

## Data Availability

The de-identified, processed data supporting the conclusions of this article are openly available in a public repository at https://zenodo.org/records/21499321 (accessed on 22 July 2026).

## Supplementary Materials

The following supporting information can be downloaded at: https://www.mdpi.com/article/10.3390/s26165219/s1, Table S1. Review of recent smartphone reliability research; Table S2. Reliability statistics (ICC, SEM%, and MDC95%) by direction, condition, and outcome measure; Figure S1. Representative trial-level anterior-posterior (AP) and mediolateral (ML) absolute-value acceleration; Figure S2. Representative trial-level anterior-posterior (AP) and mediolateral (ML) power spectral density (PSD).

## Abbreviations

The following abbreviations are used in this manuscript:

ICC	Intraclass correlation coefficient
SEM	Standard error of measurement
AARP	American Association of Retired Persons
AP	Anterior–posterior
ML	Mediolateral
COM	Center of mass
COP	Center of pressure
FT	Feet together
TAN	Tandem
SL	Single leg
EO	Eyes open
EC	Eyes closed
RMSA	Root mean square acceleration
PSD	Power spectral density
