# Supplementary material for: Reliability of a Home-Based Smartphone Balance Assessment in Healthy Middle-Aged and Older Adults
Source: Sensors (Basel). 2026 Aug 18;26(16):5219. doi: 10.3390/s26165219 (PMC13517357; doi:10.3390/s26165219)
Supplement: Supplementary file 1 [file sensors-26-05219-s001.zip › sensors-4422586-supplementary.pdf]

Table S1. Review of recent smartphone reliability research.

Study	Device	Population	Setting	Task	Design	Key Reliability Finding
Hussain & Wright [22]	iPhone 14, belt-mounted at L5, oriented horizontally	n = 22 adults (mean 43 years)	Lab; single session, researcher-controlled remotely	Quiet bipedal stance (Romberg-type), eyes open/closed × firm/foam surface	Single session; 3 repeated trials (same-day, same-session reliability)	Excellent same-day trial-to-trial reliability (ICC(3,1) > 0.90 in all conditions); good–excellent for averaged sway area/velocity (ICC(3,k) = 0.84–0.99).
Rodrigues et al. [23]	Android app, strapped to lumbar spine (L3–L5)	n = 26 healthy adults (mean 31 years)	Lab; force-platform concurrent-validity study	Bipedal stance eyes open, bipedal eyes closed, unipedal eyes open	Same session; ICC in a subsample (n = 14); not multi-day test–retest	2 of 12 parameters showed good-to-excellent reliability: total displacement (ICC = 0.81) and mediolateral RMS (ICC = 0.91)
Atalay & Kızıldoğan [24]	Android phone (Physics Toolbox Suite) fixed to a BOSU ball, not worn	n = 22 university students (mean 20.3 years)	Lab; researcher physically supported participant	Bipedal stance on an unstable BOSU ball, eyes open	3 sessions at 1-week intervals	Good-to-excellent reliability across sessions (ICC = 0.67–0.90 for sessions 1–2; 0.71–0.73 for sessions 1–3).
Prato et al. [25]	Android & iOS simultaneously, belt-mounted on lower back	n = 23 healthy adults (mean 22.9 years)	Lab; referenced to 8-camera motion capture	Tandem stance eyes open/closed, single-leg stance eyes open/closed	2 sessions, ~7 days apart	Good–to-excellent reliability eyes-open (ICC = 0.82–.98); poor–to-good eyes-closed (ICC = 0.31–.87), declining with task difficulty.
Calcagni et al. [26]	Android (Google Pixel, hand strap + neck-mount harness)	Healthy volunteers and neurological-disease cohorts	Lab visit; supervising lab member present	3 stances: eyes-open feet-apart, eyes-open feet-together, eyes-closed feet-together	Inter-day test–retest, ICC(2,1)	Few outcomes exceeded ICC > 0.5; sway-magnitude (RMS) measures most reliable, particularly eyes-closed.

ICC = intraclass correlation coefficient; RMS = root mean square; SEM% = relative standard error of measurement, expressed as a percentage of the outcome mean; MDC95% = minimal detectable change at the 95% confidence level, expressed as a percentage of the outcome mean; BOSU = Both Sides Utilized (Up), a domed balance-training device.

Table S2. Reliability statistics (ICC, SEM%, and MDC95%) by direction, condition, and outcome measure.

Direction	Condition	Outcome	ICC(2,1) [95% CI]	ICC(2,3) [95% CI]	SEM%(2,1)	SEM%(2,3)	MDC95%(2,1)	MDC95%(2,3)
**Anterior–Posterior**	**FT/EO**	Mean Accel (m/s²)	0.65 [0.52, 0.75]	0.84 [0.77, 0.90]	22.7%	15.0%	62.9%	41.6%
		RMSA (m/s²)	0.48 [0.34, 0.62]	0.74 [0.61, 0.83]	31.7%	22.5%	87.9%	62.4%
		Total PSD (0–10 Hz)	0.60 [0.47, 0.72]	0.82 [0.73, 0.88]	26.6%	17.9%	73.7%	49.6%
		PSD1 (0.0–0.25 Hz)	0.61 [0.49, 0.72]	0.83 [0.74, 0.89]	43.2%	29.0%	119.7%	80.4%
		PSD2 (0.25–0.5 Hz)	0.62 [0.50, 0.73]	0.83 [0.75, 0.89]	29.7%	19.8%	82.3%	54.9%
		PSD3 (0.5–2.0 Hz)	0.66 [0.54, 0.76]	0.85 [0.78, 0.90]	28.3%	18.6%	78.4%	51.6%
		PSD4 (2–10 Hz)	0.60 [0.48, 0.72]	0.82 [0.73, 0.88]	32.0%	21.5%	88.7%	59.6%
	**FT/EC**	Mean Accel (m/s²)	0.63 [0.50, 0.74]	0.83 [0.75, 0.90]	20.7%	13.8%	57.4%	38.3%
		RMSA (m/s²)	0.58 [0.44, 0.70]	0.80 [0.70, 0.87]	23.4%	15.9%	64.9%	44.1%
		Total PSD (0–10 Hz)	0.58 [0.45, 0.71]	0.81 [0.71, 0.88]	25.7%	17.4%	71.2%	48.2%
		PSD1 (0.0–0.25 Hz)	0.54 [0.40, 0.67]	0.78 [0.66, 0.86]	41.4%	28.7%	114.8%	79.6%
		PSD2 (0.25–0.5 Hz)	0.65 [0.52, 0.75]	0.85 [0.77, 0.90]	25.5%	16.8%	70.7%	46.6%
		PSD3 (0.5–2.0 Hz)	0.65 [0.52, 0.75]	0.85 [0.77, 0.90]	30.1%	19.9%	83.4%	55.2%
		PSD4 (2–10 Hz)	0.55 [0.40, 0.68]	0.78 [0.67, 0.86]	33.7%	23.2%	93.4%	64.3%
	**TAN/EO**	Mean Accel (m/s²)	0.70 [0.58, 0.79]	0.88 [0.81, 0.92]	25.9%	16.7%	71.8%	46.3%

Direction	Condition	Outcome	ICC(2,1) [95% CI]	ICC(2,3) [95% CI]	SEM%(2,1)	SEM%(2,3)	MDC95%(2,1)	MDC95%(2,3)
		RMSA (m/s²)	0.64 [0.51, 0.75]	0.84 [0.76, 0.90]	32.2%	21.4%	89.3%	59.3%
		Total PSD (0–10 Hz)	0.62 [0.49, 0.74]	0.83 [0.74, 0.89]	31.4%	21.0%	87.0%	58.2%
		PSD1 (0.0–0.25 Hz)	0.55 [0.40, 0.68]	0.78 [0.67, 0.86]	43.2%	29.9%	119.7%	82.9%
		PSD2 (0.25–0.5 Hz)	0.59 [0.46, 0.71]	0.81 [0.72, 0.88]	38.5%	26.1%	106.7%	72.3%
		PSD3 (0.5–2.0 Hz)	0.62 [0.49, 0.74]	0.83 [0.74, 0.89]	33.8%	22.5%	93.7%	62.4%
		PSD4 (2–10 Hz)	0.60 [0.47, 0.72]	0.82 [0.72, 0.89]	35.4%	23.9%	98.1%	66.2%
	**TAN/EC**	Mean Accel (m/s²)	0.78 [0.68, 0.85]	0.91 [0.87, 0.94]	24.6%	15.4%	68.2%	42.7%
		RMSA (m/s²)	0.70 [0.58, 0.79]	0.87 [0.81, 0.92]	31.7%	20.5%	87.9%	56.8%
		Total PSD (0–10 Hz)	0.61 [0.48, 0.72]	0.82 [0.73, 0.89]	36.3%	24.4%	100.6%	67.6%
		PSD1 (0.0–0.25 Hz)	0.32 [0.17, 0.48]	0.59 [0.38, 0.74]	52.0%	40.5%	144.1%	112.3%
		PSD2 (0.25–0.5 Hz)	0.41 [0.25, 0.56]	0.67 [0.50, 0.79]	53.2%	39.5%	147.5%	109.5%
		PSD3 (0.5–2.0 Hz)	0.59 [0.46, 0.71]	0.81 [0.72, 0.88]	44.9%	30.4%	124.5%	84.3%
		PSD4 (2–10 Hz)	0.59 [0.45, 0.71]	0.81 [0.71, 0.88]	36.2%	24.5%	100.3%	67.9%
	**SL/EO**	Mean Accel (m/s²)	0.64 [0.51, 0.75]	0.84 [0.76, 0.90]	35.4%	23.5%	98.1%	65.1%
		RMSA (m/s²)	0.54 [0.40, 0.67]	0.78 [0.67, 0.86]	45.9%	31.8%	127.2%	88.1%
		Total PSD (0–10 Hz)	0.58 [0.44, 0.70]	0.80 [0.70, 0.88]	40.7%	27.7%	112.8%	76.8%
		PSD1 (0.0–0.25 Hz)	0.56 [0.42, 0.68]	0.79 [0.68, 0.87]	42.4%	29.2%	117.5%	80.9%

Direction	Condition	Outcome	ICC(2,1) [95% CI]	ICC(2,3) [95% CI]	SEM%(2,1)	SEM%(2,3)	MDC95%(2,1)	MDC95%(2,3)
		PSD2 (0.25–0.5 Hz)	0.44 [0.29, 0.59]	0.71 [0.55, 0.81]	57.1%	41.5%	158.3%	115.0%
		PSD3 (0.5–2.0 Hz)	0.66 [0.53, 0.76]	0.85 [0.77, 0.91]	42.8%	28.2%	118.6%	78.2%
		PSD4 (2–10 Hz)	0.54 [0.39, 0.67]	0.78 [0.66, 0.86]	43.0%	29.9%	119.2%	82.9%
**Mediolateral**	**FT/EO**	Mean Accel (m/s²)	0.79 [0.70, 0.85]	0.92 [0.87, 0.95]	20.2%	12.7%	56.0%	35.2%
		RMSA (m/s²)	0.63 [0.51, 0.74]	0.84 [0.76, 0.90]	27.8%	18.5%	77.1%	51.3%
		Total PSD (0–10 Hz)	0.60 [0.47, 0.72]	0.82 [0.73, 0.88]	28.6%	19.3%	79.3%	53.5%
		PSD1 (0.0–0.25 Hz)	0.83 [0.76, 0.89]	0.94 [0.90, 0.96]	31.0%	19.0%	85.9%	52.7%
		PSD2 (0.25–0.5 Hz)	0.35 [0.19, 0.51]	0.62 [0.42, 0.76]	41.1%	31.6%	113.9%	87.6%
		PSD3 (0.5–2.0 Hz)	0.54 [0.39, 0.67]	0.78 [0.66, 0.86]	33.3%	23.1%	92.3%	64.0%
		PSD4 (2–10 Hz)	0.55 [0.41, 0.68]	0.79 [0.67, 0.86]	35.6%	24.6%	98.7%	68.2%
	**FT/EC**	Mean Accel (m/s²)	0.79 [0.70, 0.86]	0.92 [0.88, 0.95]	17.8%	11.1%	49.3%	30.8%
		RMSA (m/s²)	0.67 [0.54, 0.77]	0.86 [0.78, 0.91]	24.1%	15.8%	66.8%	43.8%
		Total PSD (0–10 Hz)	0.62 [0.49, 0.73]	0.83 [0.74, 0.89]	26.3%	17.6%	72.9%	48.8%
		PSD1 (0.0–0.25 Hz)	0.86 [0.80, 0.91]	0.95 [0.92, 0.97]	27.1%	16.3%	75.1%	45.2%
		PSD2 (0.25–0.5 Hz)	0.59 [0.45, 0.71]	0.81 [0.71, 0.88]	25.8%	17.5%	71.5%	48.5%
		PSD3 (0.5–2.0 Hz)	0.64 [0.51, 0.75]	0.84 [0.76, 0.90]	24.1%	15.9%	66.8%	44.1%
		PSD4 (2–10 Hz)	0.58 [0.44, 0.70]	0.81 [0.70, 0.88]	35.6%	24.2%	98.7%	67.1%

Direction	Condition	Outcome	ICC(2,1) [95% CI]	ICC(2,3) [95% CI]	SEM%(2,1)	SEM%(2,3)	MDC95%(2,1)	MDC95%(2,3)
	**TAN/EO**	Mean Accel (m/s²)	0.76 [0.67, 0.84]	0.91 [0.86, 0.94]	24.9%	15.7%	69.0%	43.5%
		RMSA (m/s²)	0.67 [0.54, 0.77]	0.86 [0.78, 0.91]	32.5%	21.3%	90.1%	59.0%
		Total PSD (0–10 Hz)	0.64 [0.51, 0.75]	0.84 [0.76, 0.90]	32.9%	21.8%	91.2%	60.4%
		PSD1 (0.0–0.25 Hz)	0.81 [0.72, 0.87]	0.93 [0.89, 0.95]	33.5%	20.6%	92.9%	57.1%
		PSD2 (0.25–0.5 Hz)	0.65 [0.52, 0.75]	0.84 [0.76, 0.90]	32.9%	21.7%	91.2%	60.1%
		PSD3 (0.5–2.0 Hz)	0.65 [0.52, 0.76]	0.85 [0.77, 0.90]	28.6%	18.9%	79.3%	52.4%
		PSD4 (2–10 Hz)	0.60 [0.46, 0.72]	0.82 [0.72, 0.88]	39.7%	26.8%	110.0%	74.3%
	**TAN/EC**	Mean Accel (m/s²)	0.74 [0.64, 0.83]	0.90 [0.84, 0.93]	25.5%	16.1%	70.7%	44.6%
		RMSA (m/s²)	0.67 [0.55, 0.77]	0.86 [0.78, 0.91]	30.6%	20.1%	84.8%	55.7%
		Total PSD (0–10 Hz)	0.51 [0.37, 0.65]	0.76 [0.63, 0.85]	39.0%	27.4%	108.1%	75.9%
		PSD1 (0.0–0.25 Hz)	0.64 [0.51, 0.75]	0.84 [0.76, 0.90]	36.7%	24.4%	101.7%	67.6%
		PSD2 (0.25–0.5 Hz)	0.41 [0.26, 0.56]	0.68 [0.51, 0.79]	44.9%	33.2%	124.5%	92.0%
		PSD3 (0.5–2.0 Hz)	0.47 [0.32, 0.61]	0.73 [0.59, 0.83]	39.0%	28.0%	108.1%	77.6%
		PSD4 (2–10 Hz)	0.51 [0.37, 0.65]	0.76 [0.64, 0.85]	42.4%	29.8%	117.5%	82.6%
	**SL/EO**	Mean Accel (m/s²)	0.75 [0.65, 0.83]	0.90 [0.85, 0.94]	30.4%	19.2%	84.3%	53.2%
		RMSA (m/s²)	0.71 [0.60, 0.80]	0.88 [0.82, 0.92]	34.4%	22.2%	95.4%	61.5%
		Total PSD (0–10 Hz)	0.71 [0.60, 0.81]	0.88 [0.82, 0.93]	36.2%	23.2%	100.3%	64.3%

Direction	Condition	Outcome	ICC(2,1) [95% CI]	ICC(2,3) [95% CI]	SEM%(2,1)	SEM%(2,3)	MDC95%(2,1)	MDC95%(2,3)
		PSD1 (0.0–0.25 Hz)	0.87 [0.81, 0.91]	0.95 [0.93, 0.97]	26.3%	15.9%	72.9%	44.1%
		PSD2 (0.25–0.5 Hz)	0.63 [0.51, 0.74]	0.84 [0.75, 0.90]	53.0%	35.2%	146.9%	97.6%
		PSD3 (0.5–2.0 Hz)	0.67 [0.54, 0.77]	0.86 [0.78, 0.91]	44.0%	28.8%	122.0%	79.8%
		PSD4 (2–10 Hz)	0.72 [0.61, 0.81]	0.88 [0.82, 0.93]	37.2%	23.9%	103.1%	66.2%

ICC = intraclass correlation coefficient; ICC(2,1) reflects single-trial reliability, ICC(2,3) reflects reliability of the 3-assessment mean. SEM% = relative standard error of measurement; MDC95% = minimal detectable change at the 95% confidence level, expressed relative to the outcome mean (MDC95% = 1.96 × √2 × SEM%); CI = confidence interval. FT = feet together; TAN = tandem; SL = single leg; EO = eyes open; EC = eyes closed.

Figure S1. Representative trial-level anterior-posterior (AP) and mediolateral (ML) absolute-value acceleration

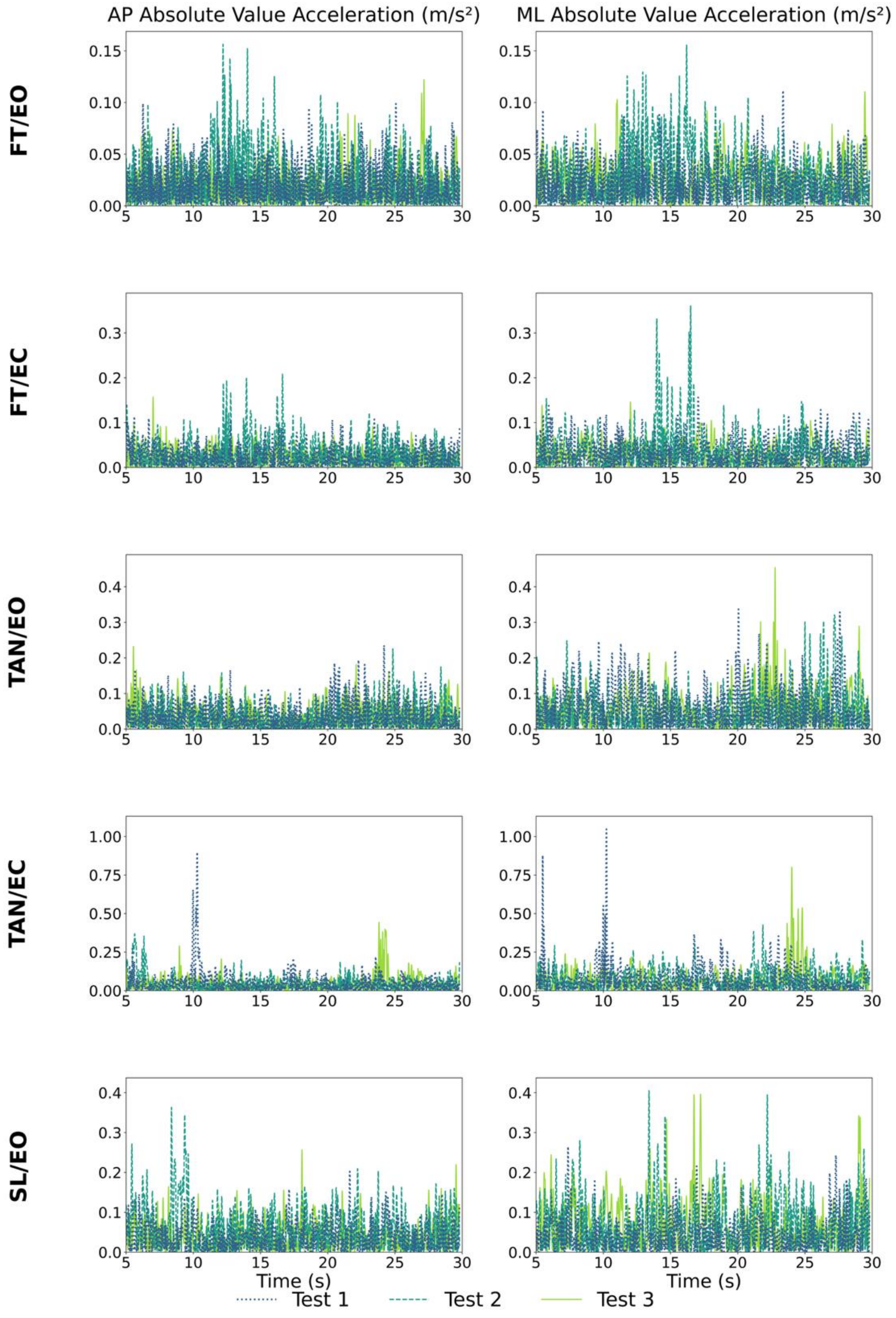

Traces for one participant (Subject 610) across all three weekly assessments (Test 1, 2, 3), by stance condition. Traces reflect the 25-second analysis window used for outcome calculation, following removal of the initial 5 seconds of each 30-second trial. FT: feet together; TAN: tandem; SL: single leg; EO: eyes open; EC: eyes closed.

Figure S2. Representative trial-level anterior-posterior (AP) and mediolateral (ML) power spectral density (PSD)

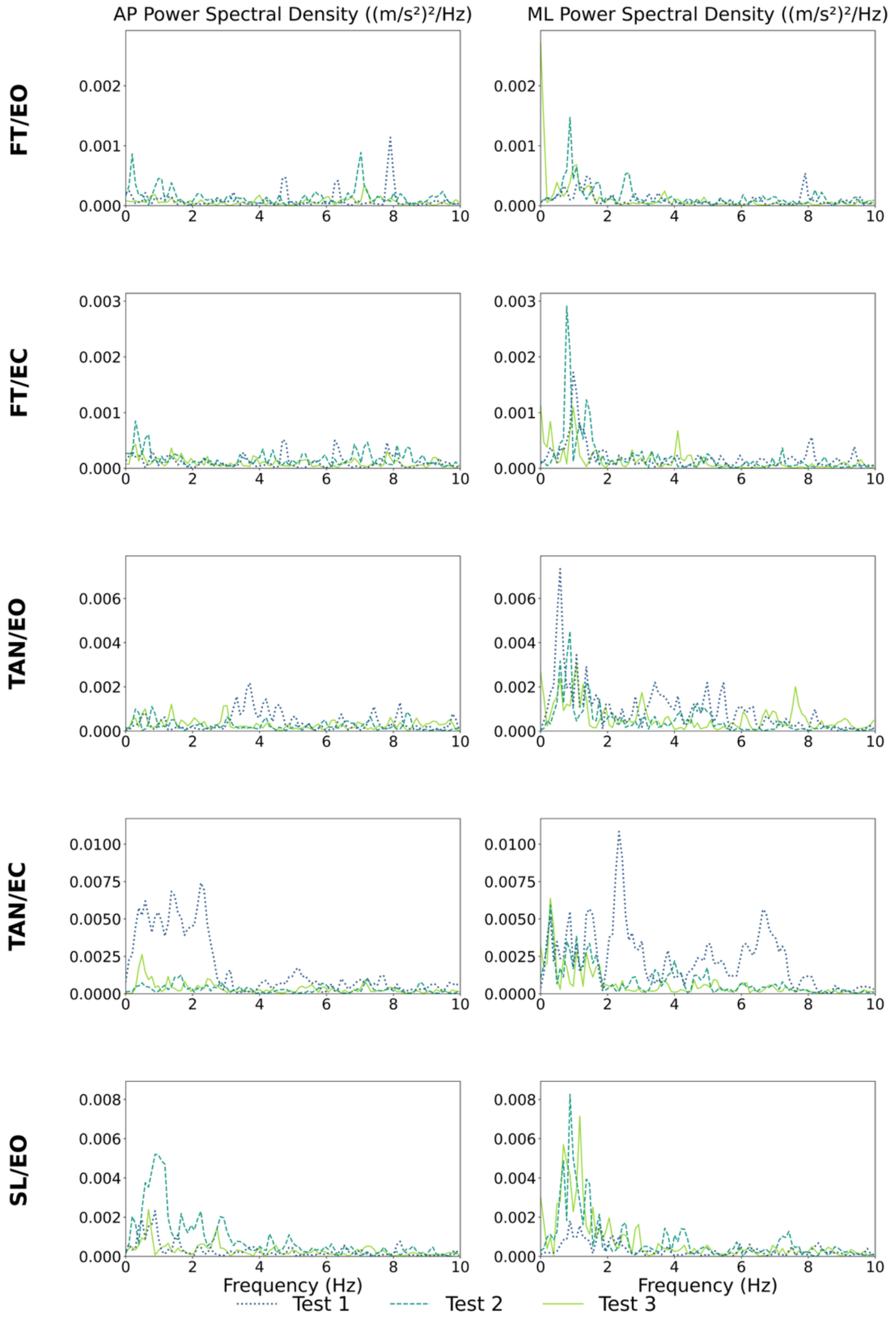

Traces for one participant (Subject 610) across all three weekly assessments (Test 1, 2, 3), by stance condition. Traces reflect the 25-second analysis window used for outcome calculation, following removal of the initial 5 seconds of each 30-second trial. FT: feet together; TAN: tandem; SL: single leg; EO: eyes open; EC: eyes closed.
